# A Behcet's Disease Patient with Right Ventricular Thrombus, Pulmonary Artery Aneurysms, and Deep Vein Thrombosis Complicating Recurrent Pulmonary Thromboembolism

**DOI:** 10.1155/2013/492321

**Published:** 2013-06-18

**Authors:** Selvi Aşker, Müntecep Aşker, Özgür Gürsu, Rıdvan Mercan, Özgür Bülent Timuçin

**Affiliations:** ^1^Department of Chest Disease, Van Training and Research Hospital, Van, Turkey; ^2^Department of Cardiology, Van Training and Research Hospital, Van, Turkey; ^3^Department of Cardiovascular Surgery, Van Training and Research Hospital, Van, Turkey; ^4^Division of Rheumatology, Department of Internal Medicine, Gazi University, Ankara, Turkey; ^5^Department of Ophthalmology, İstanbul Hospital, Van, Turkey

## Abstract

Intracardiac thrombus, pulmonary artery aneurysms, deep vein thrombosis, and pulmonary thromboembolism are rarely seen symptoms of Behcet's disease. A 20-year-old female patient was admitted for complaints of cough, fever, palpitations, and chest pain. On the dynamic thorax computed tomograms (CT) obtained because of significantly enlarged hilar structures seen on chest radiograms, aneurysmal dilatation of the pulmonary artery segments bilaterally, chronic thrombus with collapse, and consolidation substances compatible with pulmonary embolism involving both lower lobes have been observed. It is learned that, four years ago, the patient had been diagnosed with Behcet's disease and received colchicine treatment but not regularly. The patient was hospitalized. On the transthoracic echocardiogram, a thrombosis with a dimension of 4.2 × 1.6 cm was recognized in the right ventricle. On abdomen CT, aneurysmal iliac veins and deep vein thrombus on Doppler ultrasonograms were diagnosed. At the controls after three months of immunosuppressive and anticoagulant therapies, some clinical and radiological improvements were recognized. The patient suspended the treatment for a month and the thrombus recurred. We present our case in order to show the effectiveness of immunosuppressive and anticoagulant therapies and rarely seen pulmonary thromboembolism in recurrent Behcet's disease.

## 1. Introduction

Behcet's disease (BD) is a multisystem disorder presenting with recurrent buccal aphthosis, genital ulcer, and uveitis with hypopyon [1]. Pulmonary involvement in Behcet's disease is rare, occurring in 1 to 7.7% of the patients [2, 3]. Pulmonary artery aneurysms, arterial and venous thrombosis, pulmonary infarction, recurrent pneumonia, bronchiolitis obliterans organised pneumonia, and pleurisy are the main features of pulmonary involvement in Behcet's disease [4]. Cardiac involvement causes coronary artery disease, recurrent pericarditis, myocardiopathy, and endocardiac abnormalities. Intracardiac thrombus formation is very uncommon [5]. We present a Behcet's disease patient with intracardiac thrombus, pulmonary artery aneurysms, and deep vein thrombosis complicating recurrent pulmonary embolism.

## 2. Case Report

**Figure 1:**
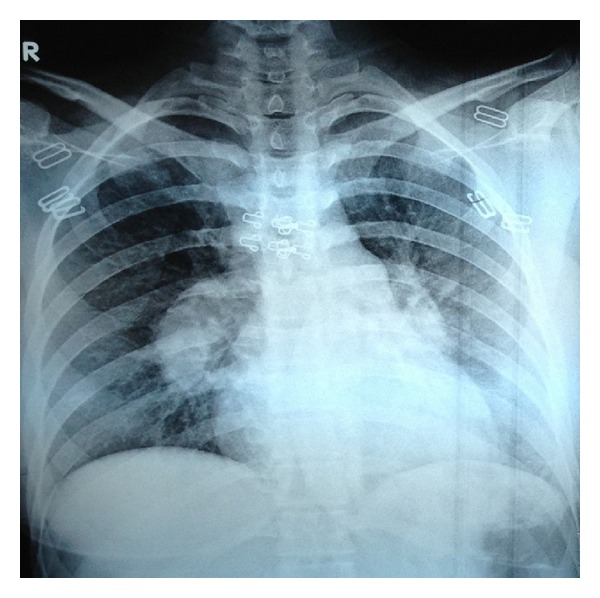
Chest radiogram demonstrating bilateral hilar enlargement and patchy infiltration.

**Figure 2:**
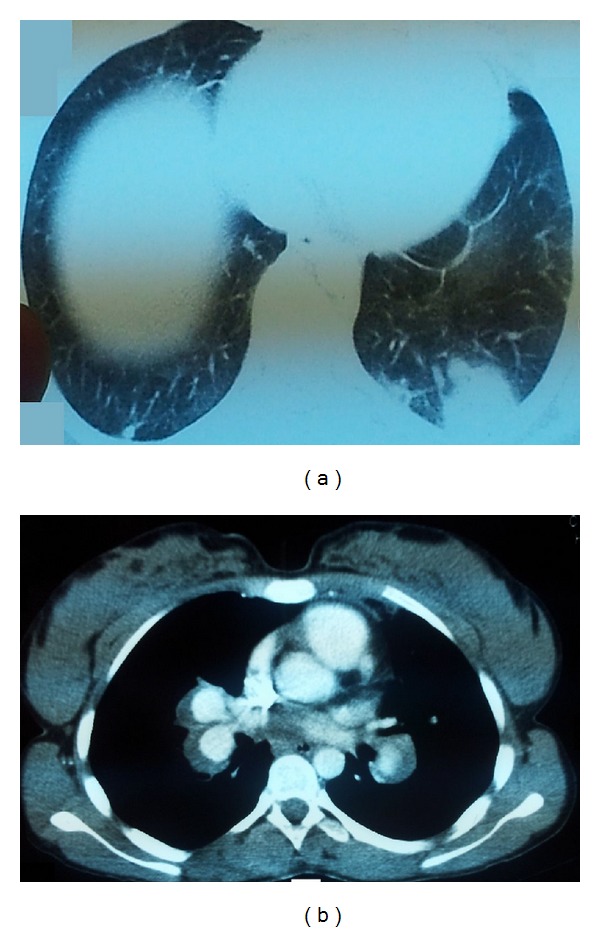
Thorax computed tomogram showing consolidated substances related to emboli in the posterobasal segment of the left pulmonary lower lobe (a), pulmonary artery thrombus and aneurysmal dilatation of the pulmonary artery (b).

**Figure 3:**
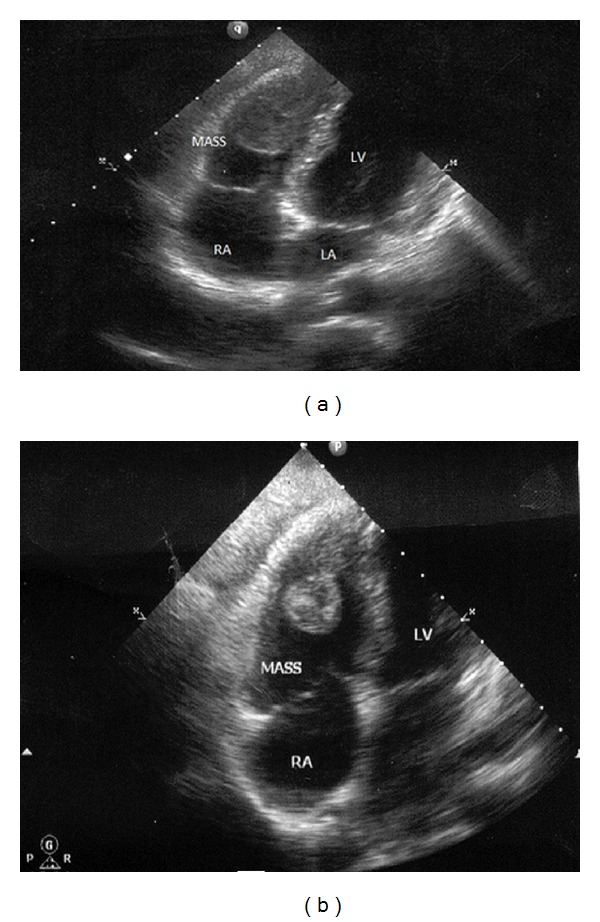
Thrombus in the right ventricle as seen on transthoracic echocardiogram (a), partial minimisation of the thrombus after the treatment (b).

A twenty-year-old woman was admitted to the hospital with complaints of cough, fever, palpitations, and chest pain. It was learned that, four years ago the patient had been diagnosed with Behcet's disease and received irregular colchicine treatment. During interviews, we learned that, he had recurrent oral and genital ulcers. On examination, there was no pathological finding except for high temperature (38.5°C). Her chest radiogram showed bilateral, hilar enlargement and peripheral but localized patchy infiltration (Figure 1). Initial diagnosis of pulmonary artery aneurysm and pulmonary emboli were especially associated with the hilar enlargement and previous diagnosis of Behcet's disease. Accordingly, dynamic contrasted CT was obtained. On dynamic thorax computed tomograms of both pulmonary arteries, aneurysmal dilatation and chronic thrombus involving all interior lmen of both pulmonary arteries were detected. Consolidated substances were determined on posterobasal segment of the left pulmonary lower lobe with a dimension of 3 × 2 cm related to, newly formed emboli (Figures 2 and 3).

**Figure 4:**
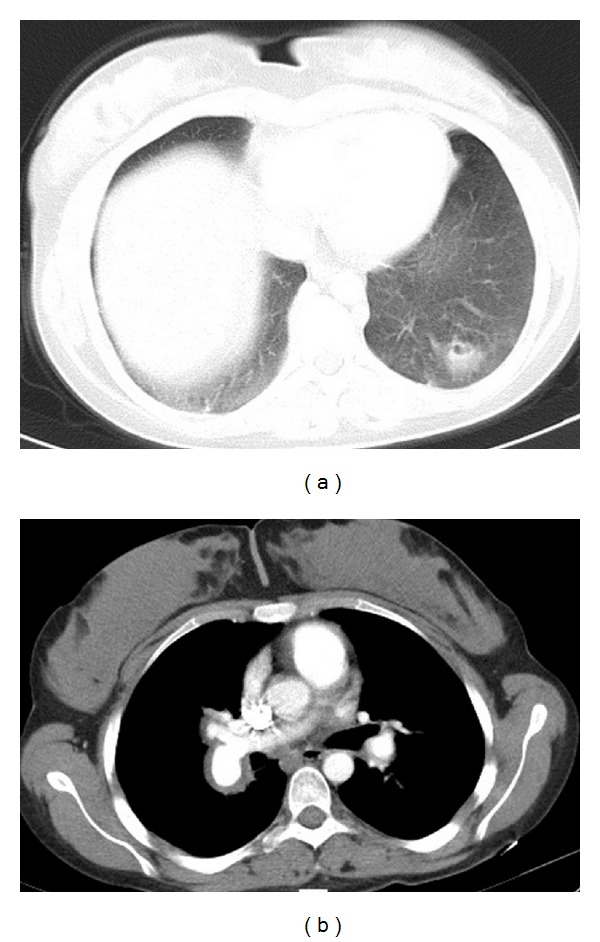
Minimisation of the consolidated substance related to the emboli on CT after the treatment (a), partial dissolution of the thrombus and minimisation of its PA diameter (b).

On concurrent transthoracic echocardiograms (TTE) a bulk with a dimension of 4.5 × 1.6 cm was determined in the right ventricle explaining symptoms of thrombus (Figure 4). On cardiac MRI, a mass lesion was detected in the right ventricle with the dimensions of 4.5 × 1.5 cm concordant with the thrombus. Thrombus was determined in the bilateral femoral veins by venous Doppler ultrasonography of the lower extremities. Hematologic parameters of the patient were within the normal limits. Thrombophilia panel was reported as normal. The patient was referred to an ophthalmologist who found evidence of active uveitis.

Pulsed immunosuppressive therapy was administered to the patient. She was quickly relieved of her symptoms after a combination therapy with methylprednisolone, cyclophosphamide, and low-molecular-weight heparin (LMWH). 

Significant improvement of the laboratory parameters of the patient was obtained (Table 1). Dynamic thorax CT was repeated at the end of the 3rd month of the treatment. Partial dissolution of the thrombi and pulmonary defects were observed. Besides, an irregular hypodense defect with irregular contours with its largest diameter comparatively regressed from 3 cm to 2 cm was observed. The diameter of the pulmonary truncus which was previously 29 mm measured 23 mm on control CT, and minimisation of the mass lesion in the right ventricle was observed (Figures 3(a), 3(b), 4(a), and 4(b)). Doppler USG demonstrated disappearance of the previously detected thrombus in femoral veins.

The patient was admitted again to the hospital on the 6th month of the treatment with complaints of leg pain, headache and palpitations. It was learned that she had quitted anticoagulant therapy 1 month ago. Doppler USG manifested evidence of recurrent DVT ischemic gliotic changes which were unobserved before being seen on brain MR angiograms. Anticoagulant treatment of the patient was repeated.

During a follow-up period of 10 months, the patient is still under treatment and doing well.

## 3. Discussion

Intrathoracic manifestations of Behcet's disease consist mainly of thromboembolism of the superior vena cava and/or other mediastinal veins, aneurysms of the aorta and pulmonary arteries, pulmonary infarct and hemorrhage, pleural effusion, and rarely, myocardial and pericardial involvement, cor pulmonale, and mediastinal or hilar lymphadenopathy [6]. Pulmonary infarction is a stage in the natural course of the disease. Pulmonary vasculitis and thromboses of pulmonary vessels may cause infarctions, focal or diffuse hemorrhages, and focal areas of atelectasis [6–8]. Although vascular involvement is seen only in 25% of the patients, it is the most common cause of mortality in Behcet's disease [6, 9, 10]. New imaging technologies, especially, dynamic thorax CT, can be helpful in the demonstration of thrombus of the systemic veins, heart and pulmonary arteries [8]. Dynamic thorax CT revealed a right ventricular thrombus in our patient. The thrombus was confirmed by echocardiography. Thromboembolism stemming from a cardiac cavity has been previously deemed to be relatively uncommon [9]. A review by Moğulkoç et al. regarding intracardiac thrombi in 25 patients with Behcet's disease was previously published [5]. The authors noted that they had seen pulmonary embolism in 13 patients (52%). In seven of these 13 patients, thrombophlebitis was observed in the major vessels which might have been the source of the embolism. Although deep venous thrombosis of the lower extremities frequently accompanies pulmonary artery aneurysms, pulmonary thromboembolism is very rare in Behcet's disease because the thrombi in inflamed veins are strongly adherent [11]. In a review study done by Houman et al. on 113 Behcet's disease patients, vein involvement had been detected in 49 patients (43.3%), and deep vein thrombus had been observed in 44 of them (38.9%). Deep vein thrombosis was more frequently observed among males (40 males and 4 females) [12]. Another review consisting of reports of Turkish authors revealed one intracardiac thrombus out of 56 (1.78%) Behcet's disease patients [13]. Recently, two Behcet's disease patients with intracardiac thrombi and pulmonary artery aneurysms have been reported [14, 15]. Luo et al. analyzed the clinical characteristics of Behcet's disease with intracardiac thrombus [16]. The data of 8 patients diagnosed with Behcet's disease with intracardiac thrombi in Peking Union Medical College Hospital from January 1990 to January 2011 were studied retrospectively. Intracardiac thrombus associated with Behcet's disease most commonly occurs in young men and usually involves the right side of the heart [16].

The pathologic mechanism of microvascular thrombus formation in vasculitis is believed to be caused by endothelial cell ischemia or disruption that leads to enhanced platelet aggregation [4, 5]. Decreased release of vascular tissue plasminogen activator has been reported in systemic and cutaneous vasculitis [9]. Impaired fibrinolysis as a result of endothelial cell injury from deposited immune complexes is another possible mechanism. Prolonged euglobulin lysis times and abnormal fibrin concentrations were found in several types of vasculitis, including Behcet's disease [6, 8]. In the present case, intracardiac thrombus, deep vein thrombosis, and pulmonary embolism were detected. Considering the possible mechanisms leading to thrombus, and recurrent emboli due to intracardiac thrombus and deep vein thrombosis, immunosuppressive and antithrombotic medications were started. Warfarin was not the preferred treatment option due to the risk of bleeding. Although the first line treatment is medical, thrombus can become massive and may demand surgical treatment. 

We presented infarct centers and new emboli centers on peripheral divisions showing chronic thrombus observed related to the repeated emboli on pulmonary arteries. During the follow-up, there was in change on thrombus divisions. Newly formed emboli were not observed and infarct centers regressed. This situation showed the effectiveness of the treatment. Our patient's pulmonary artery pressure was not high. Since pulmonary artery aneurysm decreases the load on the right side of the heart, rise in the pulmonary artery pressure might be observed in cases with severe pulmonary embolism. There was cardiac and deep vein thrombus inside the right ventricle of our patient. As these two diagnoses might cause recurrent embolisms *per se*, anticoagulant treatment needs to be used concomitantly with the immunosuppressive treatment. Some publications have asserted that these patients were subjected to bleeding episodes, and anticoagulant treatment is contraindicated for them. On the contrary, we saw that this treatment prevented occurrence of recurrent embolisms.

We have initiated methylprednisolone and cyclophosphamide combination therapy as suggested for the management of other severe forms of systemic vasculitis. We added an anticoagulant treatment into this combination. We have observed clinical and radiological improvement with this treatment. 

We kindly deemed suitable to present this case report in order to show the necessity of anticoagulant treatment to be added to the immunosuppressive therapy in such complicated cases.

## Figures and Tables

**Table 1:** Laboratory findings after and before the treatment.

	Pretreatment	Posttreatment
Hemoglobin (g/dL)	12.6	12.2
White blood cell (WBC) (/mL)	16.5	7.6
Erythrocyte sedimentation rate (ESR) (mm/h)	55	10
C-reactive protein (CRP) (mg/dL)	94	1
Fibrinogen (mg/dL)	501	290
D-dimer (ng/dL)	699	233
